# Acute Cholangitis following Intraductal Migration of Surgical Clips 10 Years after Laparoscopic Cholecystectomy

**DOI:** 10.1155/2015/504295

**Published:** 2015-03-22

**Authors:** Natalie E. Cookson, Reza Mirnezami, Paul Ziprin

**Affiliations:** ^1^Academic Surgical Unit, St Mary's Hospital, 8th Floor, QEQM Building, Praed Street, London W2 1NY, UK; ^2^Biosurgery & Surgical Technology, Department of Surgery & Cancer, Imperial College London, St Mary's Hospital, 10th Floor, QEQM Building, Praed Street, London W2 1NY, UK

## Abstract

*Background*. Laparoscopic cholecystectomy represents the gold standard approach for treatment of symptomatic gallstones. Surgery-associated complications include bleeding, bile duct injury, and retained stones. Migration of surgical clips after cholecystectomy is a rare complication and may result in gallstone formation “clip cholelithiasis”. *Case Report*. We report a case of a 55-year-old female patient who presented with right upper quadrant pain and severe sepsis having undergone an uncomplicated laparoscopic cholecystectomy 10 years earlier. Computed tomography (CT) imaging revealed hyperdense material in the common bile duct (CBD) compatible with retained calculus. Endoscopic retrograde cholangiopancreatography (ERCP) revealed appearances in keeping with a migrated surgical clip within the CBD. Balloon trawl successfully extracted this, alleviating the patient's jaundice and sepsis. *Conclusion*. Intraductal clip migration is a rarely encountered complication after laparoscopic cholecystectomy which may lead to choledocholithiasis. Appropriate management requires timely identification and ERCP.

## 1. Background

Cholecystectomy represents the gold standard for definitive treatment of symptomatic gallstones. Estimated 57,000 cholecystectomies were performed in the UK in 2012 [1] and around 600,000 are performed in the United States annually [2]. Approximately 90% of these are performed laparoscopically in the modern era, and this can involve a 3-port, 4-port, or single-port laparoscopic technique [3]. Irrespective of approach, following dissection of Calot's triangle, the majority of surgeons opt for clipping the cystic duct (CD) before dividing it. Surgical clip migration following cholecystectomy was first described by Walker et al. in 1979 [4], and since then a limited number of case reports have described this rare complication [5, 6]. The precise pathophysiological mechanism responsible for clip migration remains uncertain, though it is understood that the migrated clip(s) can serve as a nidus for stone formation (coining the term “clip cholelithiasis”). Here we describe the case of a patient who presented with acute cholangitis secondary to intraductal migration of surgical clips 10 years after laparoscopic cholecystectomy.

## 2. Case Presentation

**Figure 1:**
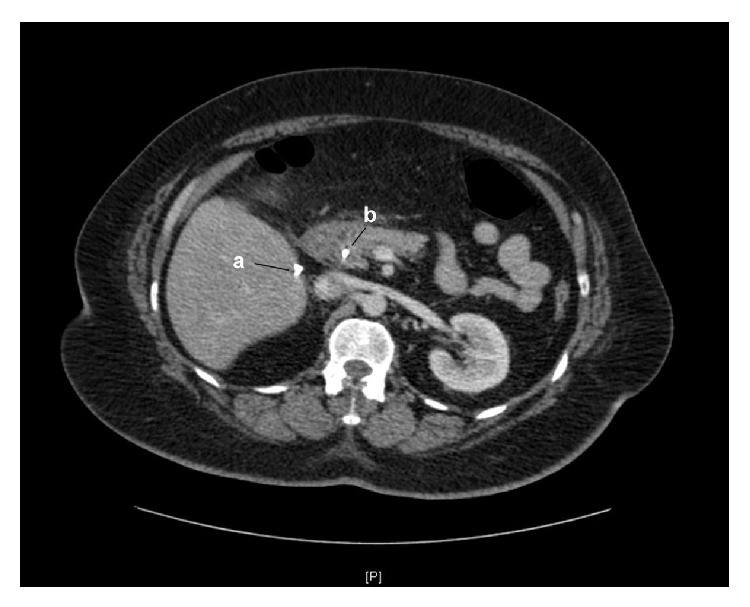
CT abdomen demonstrating (a) hyperdense material corresponding to surgical clip(s) in gallbladder fossa and (b) hyperdense material in the distal CBD.

**Figure 2:**
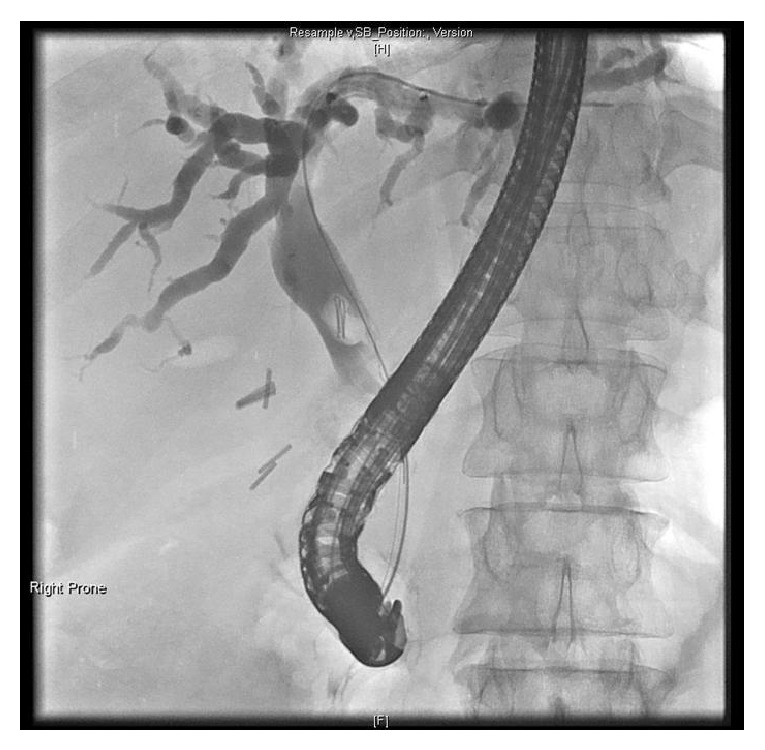
Images obtained at ERCP demonstrating intra- and extrahepatic ductal dilatation secondary to an occluding stone in the distal CBD, passing through the pancreatic head, formed around two migrated surgical clips.

A 55-year-old female was brought in by ambulance to Accident & Emergency with severe abdominal pain, nausea, vomiting, and overwhelming sepsis. The patient was visibly jaundiced at presentation. Physical examination revealed marked tenderness in the right upper quadrant and the patient was found to have a temperature of 38.5° Celsius, with associated tachycardia (HR ~120 bpm) and hypotension (systolic BP ~80 mmHg). The only feature of note in her past medical history was an uncomplicated laparoscopic cholecystectomy some 10 years earlier. Initial laboratory indices were as follows: WBC 14 × 10^9^/L, ALT 78 IU/L, ALP 242 IU/L, and total bilirubin 97 umol/L. After fluid resuscitation the patient was commenced on broad spectrum antibiotics and transferred to the high dependency unit (HDU) for vasopressor support. The working diagnosis at this stage was biliary sepsis, likely secondary to a retained stone in the common bile duct. Computed tomography (CT) imaging of the chest, abdomen, and pelvis revealed a moderate right sided pleural effusion and evidence of intra- and extrahepatic ductal dilatation. Hyperdense material was noted in the lower common bile duct, and a surgical clip was visible in the gallbladder fossa (Figure 1). The patient had mild coagulopathy corrected with 10 mg IV vitamin K and urgent endoscopic retrograde cholangiopancreatography (ERCP) was arranged. ERCP with sphincterotomy and balloon trawl was performed and it resulted in removal of a stone from the lower CBD formed around two migrated surgical clips (Figure 2). This resulted in a swift improvement in the patient's condition and progressive normalisation of previously deranged liver function tests. The patient was discharged from hospital 13 days after initial presentation.

## 3. Discussion

Although rare, clip migration following laparoscopic cholecystectomy has been described in a number of case reports and should be recognised as a potential late complication after surgery. Though first described as a potential postcholecystectomy complication in 1979 [4], the exact incidence of recurrent stone formation around metallic clips remains unknown. The precise mechanism responsible for clip migration remains unclear, though it has been suggested that compression of the clipped CD stump over time by adjacent structures (in particular the liver) can lead to invagination of the CD and clips into the CBD [7]. It is thought that the inverted CD stump eventually undergoes necrosis, presumably due to local pressure effects that follow inversion, allowing the clips to fall away into the CBD lumen, where they can serve as a focal point for gallstone formation [7]. Operative factors are likely to play an important role and logically a short CD stump with clips applied in close proximity to the CD/CBD junction may result in a greater risk of subsequent clip migration. Furthermore, accurate clip placement during surgery may prevent loosening, dislodgement, and ultimate migration of clips. Some authors have suggested that the use of absorbable suture material in favour of metallic clips may have a role in preventing foreign-body associated stone formation [8]. However cases of stone formation around absorbable material have also been described [9]. It should be noted that biliary clip migration is not restricted to internalisation into the CBD; poor clip application to the CD can lead to clip dislodgment and resultant biliary peritonitis [10]. In addition, a review of the literature reveals that clip migration after cholecystectomy can also lead to other gastrointestinal complications; for example, cases of clip migration into the duodenum have been described which have led to duodenal ulceration [11].

In our opinion, thorough skeletonisation of the CD and distal application of the minimum number of metallic clips/ligatures are measures that are more likely to reduce the likelihood of CD stump invagination and may reduce the risk of inadvertent clip migration. Although this is a rare cause of cholangitis after cholecystectomy of unproven pathogenesis, it should be recognised early with correct imaging and treated with ERCP and stone extraction.

## 4. Learning Objectives

1. “Clip cholelithiasis” is a rarely encountered complication following laparoscopic cholecystectomy which can lead to obstructive jaundice.
2. The precise pathophysiological mechanism by which surgical clips migrate from the original site of application remains uncertain.
3. The time interval between cholecystectomy and clip migration can vary, and symptoms may not manifest until years after the initial procedure.

## References

[1] Association of Upper Gastrointestinal Surgeons (AUGIS) (2013). *Gallstones Commissioning Guide*.

[2] Chong V. H., Chong C. F. (2010). Biliary complications secondary to post-cholecystectomy clip migration: a review of 69 cases. *Journal of Gastrointestinal Surgery*.

[3] Csikesz N. G., Singla A., Murphy M. M., Tseng J. F., Shah S. A. (2010). Surgeon volume metrics in laparoscopic cholecystectomy. *Digestive Diseases and Sciences*.

[4] Walker W. E., Avant G. R., Reynolds V. H. (1979). Cholangitis with a silver lining. *Archives of Surgery*.

[5] Photi E. S., Partridge G., Rhodes M., Lewis M. P. N. (2014). Surgical clip migration following laparoscopic cholecystectomy as a cause of cholangitis. *Journal of Surgical Case Reports*.

[6] Kager L. M., Ponsioen C. Y. (2009). Unexpected bile duct stones formed around surgical clips 4 years after laparoscopic cholecystectomy. *Canadian Journal of Surgery*.

[7] Kitamura K., Yamaguchi T., Nakatani H. (1995). Why do cystic duct clips migrate into the common bile duct?. *The Lancet*.

[8] Oh H. J., Jung H. J., Chai J. I. (2003). A case of common bile duct stone developed due to a surgical clip as a nidus: an experience of successful management by endoscopy. *The Korean Journal of Gastroenterology*.

[9] Maeda C., Yokoyama N., Otani T. (2013). Bile duct stone formation around a nylon suture after gastrectomy: a case report. *BMC Research Notes*.

[10] Yao C. C., Wong H. H., Chen C. C., Wang C. C., Yang C. C., Lin C. S. (2001). Migration of endoclip into duodenum. A rare complication after laparoscopic cholecystectomy. *Surgical Endoscopy*.

[11] Samim M. M., Armstrong C. P. (2008). Surgical clip found at duodenal ulcer after laparoscopic cholecystectomy: report of a case. *International Journal of Surgery*.

